# Fight or flight—intensive care nurses’ decisions to resign following the COVID-19 pandemic: a phenomenological hermeneutical study

**DOI:** 10.1186/s12912-025-02956-7

**Published:** 2025-04-01

**Authors:** Anna Slettmyr, Anna Schandl, Maria Arman, Karin Hugelius

**Affiliations:** 1 Faculty of Medicine and Health, Örebro University, Örebro, Sweden; 2 Department of Neurobiology, Care Sciences and Society, Karolinska Institutet, Huddinge, Stockholm, Sweden; 3 Department of Clinical Science and Education, Södersjukhuset, Karolinska Institutet, Stockholm, Sweden

**Keywords:** Intensive care nurses, COVID-19, Ethics, Phenomenological hermeneutical, ICU, Nursing management

## Abstract

**Background:**

Many intensive care unit (ICU) nurses who were crucial to the frontline response during the COVID-19 pandemic left their employment during or after the pandemic. Studies exploring the experiences of these nurses are lacking. The aim of this study was to explore ICU nurses’ course towards making the decision to resign from work in the ICU following the COVID-19 pandemic.

**Method:**

Advertisements on social media and a snowball sampling-inspired method were used to recruit 11 nurses from hospitals around Sweden who worked in an ICU during the pandemic and who then left employment. The participants were interviewed individually via telephone, online or in-person. An interview guide with a few open-ended questions was used to capture the nurses’ narratives. The data were analysed using a phenomenological hermeneutical method.

**Results:**

The nurses were tangled in paradoxes, described as three themes: ‘*To give it all and yet feel insufficient’*, ‘*To experience togetherness and yet feel lonely*’ and ‘*To prioritise others and yet need to eventually prioritise oneself’*. The decision to end their employment was ambivalent but necessary, made with relief and no regrets, but with sorrow. During this decision-making process, there may have been a window of opportunity during which nursing management or the health care service might have influenced the outcome.

**Conclusion:**

The ICU nurses’ decision to resign was influenced by a tangle of challenging paradoxes that entailed ambivalence. The course to the decision to resign was marked by hesitancy. While it is important to understand and support nurses’ willingness to care for patients during a crisis and to acknowledge their suffering as it relates to their professional efforts, it is also essential to address their individual struggles and needs.

**Supplementary information:**

The online version contains supplementary material available at 10.1186/s12912-025-02956-7.

## Background

Nurses in intensive care (ICU) were on the frontline of the COVID-19 pandemic. The pandemic impacted both physical and psychological aspects of their lives, resulting in growing concerns about the safety and health of nurses’ as well as the wellbeing of their families [1]. Nurses showed a great willingness to care for patients [2–4] and made altruistic sacrifices that caused them subsequent suffering and health problems [5], such as depression, post-traumatic stress disorder, physical symptoms [6, 7] and compassion fatigue [8]. Working long days in a high-risk clinical environment with inadequate protection, staff shortages and an immense workload increased their risk of burnout [8, 9]. These nurses showed a great sense of duty to provide care and worked hard; many also left the ICU.

The shortage of nurses worsened as the pandemic continued [4]. Factors affecting nurses’ intention to leave employment during the pandemic were related primarily to infection, the pandemic itself, and to work environment issues such as one’s own risk of becoming infected, a lack of personal protection equipment and long working hours. These factors affected social relations and caused high stress levels, which were exacerbated by a lack of administrative or managerial support. All of these factors could potentially have been avoided [10].

Several studies have explored nurses’ experiences of caring for patients during the COVID-19 pandemic, with the result showing what a demanding and challenging situation it was for them [5, 11, 12]. Research has also explored the factors that affected nurses’ intention to leave their workplace in relation to the pandemic [3, 10, 13]. However, only one quantitative survey was found to have investigated ICU nurses’ motives to leave the ICU, although it did not primarily address these motives in relation to COVID-19 [14]. Hence, to our knowledge no studies have thus far explored the experiences of ICU nurses who not only intended to resign, but actually did resign during the COVID-19 pandemic. Such research, which could lead to deeper knowledge about the phenomenon, may yield important insights for both nurses and nursing managers for the future, as well as for the health care service as an organisation.

## Methods

### Aim

This study aimed to explore ICU nurses’ course towards making the decision to resign from work in the ICU following the COVID-19 pandemic.

### Design

A qualitative design was applied to explore the complex phenomenon of nurses’ life choices during a global pandemic. Aiming to elucidate the meanings of life world phenomena through nurses’ narrated lived experiences of caring for patients during a pandemic, the analysis followed a phenomenological hermeneutical method [15, 16] inspired by the theory of interpretation presented by Ricoeur [17]. The study conformed to the principles of the Declaration of Helsinki [18] and is reported following the consolidated criteria for reporting qualitative research (COREQ) [19].

### Theoretical departure

This study departs theoretically from the philosophies of Knud Ejler Løgstrup [20] and the tradition of caring related to the theories of Kari Martinsen [21–23]. As humans we are interdependent and vulnerable; these essential human conditions mean that we must place our trust in, and hand ourselves over to, our fellow human beings. When encountering others’ vulnerability and suffering, the sovereign life expressions evolve. According to Løgstrup [20], we always carry a part of our fellow human beings in our hands. The theory of the ethical triad, consisting of (1) sovereign life expressions, (2) ethical demands and (3) interactions with relative culture-bearing norms [21, 22], forms the basis for interpretation and discussion of the phenomena of interest in this study. Sovereign life expressions such as mercy, trust and hope appear spontaneously in our encounters with fellow human beings. Grounded in interdependence, the ethical demand to respond to vulnerability and suffering of others arises when encountering fellow human beings. It is an unspoken demand to care for what is given to us in these situations [22]. We experience this demand to respond even if it disturb or unsettles our existence [20]. Living our lives as relational and interdependent individuals means taking care of the life placed in our hands, which is also applicable to caring and the nursing profession [21].

### Participants

The inclusion criteria for the study were (1) intensive care nurses directly involved in the care of COVID-19 patients during the pandemic (2) who then left ICU employment.

According to the challenges involved in finding participants who were no longer employed in an ICU, recruitment was accomplished primarily through advertisements on social media and a snowball sampling-inspired method that involved asking enrolled participants and employed ICU nurses to distribute information about the study to former colleagues. Potential participants were directed to a homepage where they could read the full study information and sign a consent form. On the homepage, potential participants could enter basic demographic information about their sex, age and work experience, and whether they wanted to be interviewed via telephone, online or in person. One researcher (ASl) contacted the potential participants and set up arrangements for the interviews.

Eleven Swedish nurses who specialised in intensive care, which included some with an additional specialisation in anaesthetic care, participated. These consisted of four men and seven women aged between 32 and 59 years (median 43 years). They had between four and 30 years of experience working in intensive care (median 10.5 years) and had worked as general nurses for between one and 15 years (median five years) before doing their specialisation. Seven of the nurses had worked in ICUs in a university hospital and four in a regional hospital. The participants found out about the study from advertisements on social media (n = 4), by coincidence on the internet (n = 1), or through former colleagues who had received the information from the researchers (ASl and ASch) (n = 6).

### Data collection

Individual interviews with the participants were conducted from August 2023 to March 2024. All interviews lasted approximately 60 min, except for two that lasted about 100 min (50–105 min). The interviews were conducted on the telephone (n = 5), as online interviews via Zoom (n = 4) or as in-person meetings (n = 2). The nurses were informed about the possibility of withdrawing from the study at any time and about sharing only the experiences they felt comfortable sharing. An interview guide (see Supplementary File 1) developed specifically for this study and informed by previous research and the research team’s qualitative experiences was used to capture the nurses’ narratives of their lived experiences. This guide, which contained a few open-ended questions, aimed to explore the course towards making the decision to resign from their employment in the ICU. The interviews were conducted by one of the researchers (ASl) and were audio recorded. All interviews were transcribed verbatim by a transcription agency.

### Data analysis

The data were analysed using a phenomenological hermeneutical method in accordance with Lindseth and Norberg [15], and the analysis was conducted in three steps: naïve understanding, structural analysis and formulation of a comprehensive understanding. The 11 interviews were initially read separately by all four members of the research team. Thereafter, the researchers met and reflected together on the interviews, one at a time, to obtain a collective reflection, lasting about 45 min each. Then, the first naïve understanding was formulated as a basis for a structural analysis [15] that started with the extraction of meaning units relevant to the study’s purpose. Those meaning units were reflected upon, primarily by two of the authors (ASl and KH), and condensed into subthemes that collected essential meanings for the course of making the decision to leave ICU employment. The subthemes were then reflected upon, and the phenomena that revealed themselves were identified and formulated as themes. The subthemes and themes were repeatedly validated in relation to the naïve understanding, and vice versa, due to the hermeneutic circle [16]. Finally, a comprehensive understanding was formulated as an interpretation of the findings as a whole in relation to the naïve understanding and the theoretical departure point–that is, the theories of Løgstrup and Martinsen. To strengthen the validity and credibility of the analytic process, the findings were discussed repeatedly amongst all the authors. In accordance with the phenomenological hermeneutic method, the findings are presented in order of the analysis process: (1) the naïve understanding, (2) the structural analysis and, finally, (3) a comprehensive understanding [15, 16].

## Results

### Naïve understanding

The initial self-evident approach shown by the nurses in this study in caring for patients, as well as their great willingness to help their fellow human beings, slowly changed to a need to save themselves. In pushing their physical and mental boundaries, working hard and putting their own needs on hold, a feeling of insufficiency was present. This resulted in disappointment in themselves, exhaustion, and a sense of failure when they were unable to handle the situation. However, also experienced were feelings of gratefulness for and pride in being part of the event and contributing to the management of the crisis. Collegiality, or the feeling of ‘being in this together’, greatly bolstered their endurance. However, at the same time, experiences of loneliness and abandonment were expressed, and those feelings consisted mostly of disappointment with the lack of support from and preparedness for such a situation amongst management, leading the nurses to being left to solve difficult problems and challenging situations on their own. Struggling with one’s own moral inner compass when dealing with ethical and caring challenges, combined with encountering a huge amount of suffering and not being able to provide care to the desired standard, resulted in frustration and hopelessness. In the end, a decision had to be made weather to stay and fight, with the risks of severe consequences for oneself, or to leave employment to save oneself. When the decision to leave was made, it was a relief, but it also felt like a betrayal to abandon one’s fellow human beings (i.e. patients and their families as well as colleagues).

### Structural analysis

An overview of the themes and subthemes is presented in Table 1.

**Table 1:** Structural analysis—themes and subthemes

Theme	Subthemes
To give it all and yet feel insufficient	Self-evident to care in the situation & took it as a challenge Knowing my value as an ICU nurse Feeling proud & grateful Ambivalence towards what the situation demanded Feeling insufficient & guilty for one’s own shortcomings Feelings of regret, bad conscience & hopelessness
To experience togetherness and yet feel lonely	Community and loyalty with colleagues an important support and a motivator to care Taking responsibility for competence in the workplace Support from managers of great importance Lack of support from the organisation & not given the right conditions to care A feeling of loneliness No appreciation; rather a feeling of being used
To prioritise others and yet need to eventually prioritise oneself	Balancing priorities Immense impact on the physical, psychological and personal aspects of one’s life Coping with suffering and death Protecting important caring values and one’s own moral boundaries Reaching a breaking point A relief to leave the ICU but a loss of the expected professional future Ambivalent approach to care in the event of a new pandemic

#### To give it all and yet feel insufficient

When the pandemic struck Sweden, nurses demonstrated an innate dedication and a profound willingness to care for patients. This commitment was the result of their choice of profession, a desire to help people, a sense of responsibility towards others, a readiness to contribute and a confidence in their vital competence as ICU nurses:

… after all, I have a very high degree of sense of responsibility towards the patients. So, you have to … they are completely in our hands (N6).

The situation was seen as a mostly positive challenge to undertake, but the feeling of not having a choice was also present. Performing the work out of a sense of duty, sometimes with some hesitation, led to reflections on not being willing to die for one’s work. Although the situation was described as tiresome, there was no other choice but to persevere and handle the challenges that arose. In showing what could be achieved, a sense of pride and satisfaction emerged from being part of society’s response to the crisis and making a meaningful difference: *‘For a short period, it felt like the world was looking at us and appreciated what we were doing’* (N4).

The feeling of pride in their own competence and the opportunity to contribute was exemplified by the statement, *‘It takes a long time to be an experienced ICU nurse’* (N3). Caring for patients during the pandemic was a valued experience that encompassed gratitude and satisfaction, despite the hope of never having to experience it again.

As time passed, the initial excitement and interest waned, giving way to a sense of teetering on the edge, never fully able to relax or feel secure. This created ambivalence towards the demands of the situation. High expectations to manage the circumstances led to self-disappointment and guilt, especially when the heavy workload hindered their own standards and expectations of good care:

… I felt a lot of anxiety (…) I had my patients to whom I had to give good care, and we had to try to save their lives while they were ‘actively’ trying to die here. But I have so many patients and so little help that I don’t have time to do the most basic things. (…) … given that feeling, you couldn’t give what you’re used to (N4).

Feelings of shame for not prioritising their own well-being, along with regrets for not asking for more help, were expressed. Further, the participants reflected on whether this might have protected their well-being and increased their chances of staying employed in the ICU context.

Experiences of being moody with colleagues and others at work and in private life were described. The lack of strength to give that little extra care to patients and their families also led to feelings of shame. Additionally, there were feelings of guilt over contributing to patient complications and a fear of having made mistakes that could have negatively affected the patients. This included feelings of guilt for not providing family members with the support they needed:

… retrospectively, we could have done [*things*] differently, as with relatives, for example. That is what hurts me most—that we maybe did [*something*] wrong with that (N6).

Caring with no end to the pandemic in sight and with the situation dragging on indefinitely was difficult and resulted in feelings of hopelessness.

#### To experience togetherness and yet feel lonely

An impressive community emerged from the severe situation. Being in it together with colleagues, and supporting one another, was of utmost importance in coping with the crisis:

That was probably the most amazing thing in all the tragedy: that we on our work team became so incredibly united and had such incredible respect and humility for the situation and for each other. So, I think that was one of the parts that I take with me as positive: how important it was to support each other (N10).

Loyalty and solidarity with colleagues and sharing the burden were key motivators for working. The commitment to not abandon colleagues and to support each other was paramount, even to the extent of going to work with a fracture and leg cast. Collegiality fostered great responsibility for the workplace, with extra shifts being taken and tasks being done ‘the right way’. Conversely, guilt was experienced during sick leave and grew stronger when thoughts of resigning began and eventually led to the decision to leave.

Support and solidarity from managers, along with a feeling of trust in the management, were highly valued. However, a lack of support from some managers was described, with instances of absenteeism and disinterest in daily clinical work challenges. This resulted in feelings of loneliness, despite the appreciated collegiality and community with colleagues. Further, disappointment increased when better organisational preparedness for such a situation was expected but not realised:

… realising no help will arrive from ‘above’. No one is going to help … (…), so it is me and my colleague’s responsibility to try to make the impossible possible. (…) I felt very abandoned and frustrated (N6).

Employees who were moved from their usual workplace to other units described a feeling of being a ‘guest worker*’* who has been left out of the new community. Being moved around different units was described as ‘*you only felt like a pawn in the game’* (N4). However, upon returning to one’s original workplace, a sense of exclusion from the ordinary community was experienced due to not having worked with their colleagues for a time.

The conditions necessary for providing quality care were lacking. In many situations, the right tools for handling challenges were missing. There was often no time for collegial feedback, the fundamentals of care or training new colleagues. The situation demanded significant responsibility with little support: ‘*And then this frustration started to grow, like, I cannot, whatever I do, I cannot help the patients’* (N5). Attempts to raise issues and file structured error reports were ignored, and reports about deficiencies in care received no responses. There was also a growing impression of no longer sharing with one’s managers the core values of caring.

The lack of appreciation and support, along with not being recognised as unique individuals, was experienced deeply. Being asked to cover for colleagues on sick leave, even when one was drained or just returning from one’s own sick leave, was perceived as ‘*stepping on those already lying down’* (N3), which also created a sense of being used. Not being included in organising their own working situation and being left out of decisions about when, where and how much to work, resulted in feelings of exclusion and even of being a serf.

Another kind of loneliness arose when discussing workplace conditions with people outside of work. The reluctance of others to listen to or believe the experiences shared, especially within the context of the pandemic, further deepened this sense of loneliness.

#### To prioritise others and yet need to eventually prioritise oneself

Working tirelessly to support patients, families and colleagues often led to prioritising others’ well-being far more than one’s own:

… I really worked without coming up for air and far below the surface. So, actually, when I look at it now, personally, I think that it’s absolutely incredible that I managed … (…). Sometimes, you have more inherent strength than you think when faced with challenges like this … (N11).

Participants also described the struggle to retain important caring and ethical values and to follow one’s inner moral compass while standing up for one’s fellow human beings in ethically challenging situations. Priorities had to be made, both for oneself but also by physician colleagues, which often challenged that moral compass. While many priorities were perceived to be reasonable in the circumstances, others were seen as ethically challenging, unreasonable, unclear, unfair and sometimes even condemnable:

We have abused this woman [i.e. *treated her too long and prolonged her suffering*], and finally, we are about to stop. (…) And there I had enough. I felt that I could not be part of this anymore (N1).

Reflections emerged on whether stricter criteria for patients’ admittance to the ICU could have preserved the health and well-being of the ICU nurses, but such thoughts were considered too ethically repugnant to fully contemplate. While the most important goal remained the survival of the patients, the need to prioritise one’s own well-being was also raised.

However, the effort came with significant personal costs and physical, psychological and social consequences. For instance, there was a loss of control over one’s life situation and a decline in empathy and engagement with family and children, leading to sacrifices in time spent with friends and family. Working harder than ever before caused the body to ‘shut down’ due to inadequate rest and recovery time, adversely affecting sleep quality. Physical impacts from that period, included reduced strength to handle work situations, impaired cognitive ability, enormous fatigue and severe hand eczema. Normal coping strategies for recovery, such as sleep and physical activity, no longer worked, and the impact on the body ultimately became too severe to handle.

The perception of caring for ‘bodies’ emerged since many patients had similar features and the absence of family visits led to patients becoming ‘anonymous’. The impersonal nature of care and the need to detach from patients had both professional and personal consequences. One nurse described coming home to find her husband sleeping in bed, lying on his stomach in the same prone position and with features similar to many of the ICU patients, which evoked unpleasant feelings of recognition.

Experiencing significant suffering and death along with witnessing how innocent people were affected by the lack of preparation for a pandemic situation was challenging. Many stories were shared about patients and families being unable to say their farewells in person, but rather, due to visiting restrictions, through digital means, glass windows or while wearing full personal protective equipment. There were efforts to retain one’s dignity in such situations. Memories of patients dying alone and bereaved children were still deeply affecting:

I still have pictures in my head of three kids … (…), I see them leave the ICU through the hospital corridor and disappear around a corner, holding each other, and I know they have lost both their mother and father. Memories of situations like that still move me when I think about them (N10).

Such experiences, combined with the compromise of patient safety, contributed to reaching a breaking point where it became impossible to accept or justify the conditions, the care provided or the decisions made. To cope with these situations, emotionally distancing oneself from patients led to reduced empathy, which was expressed as the price to pay to survive the circumstances:

… before, maybe I could balance the suffering with the fact that it was exciting to do this, but now it wasn’t. I was more affected by the patients’ suffering. (…) It didn’t work. I just got angry. I couldn’t find any way to deal with it other than running away (N1).

At one point, a decision to stand up for one’s own needs became necessary when sacrificing personal health and life was no longer justifiable. Several coping strategies were attempted to handle the situation, but when these did not work, the urgency to resign grew stronger. Some managed to leave before reaching the point of burnout. Others, guided by their moral compass, resigned abruptly after conflicts with managers about the quality of care. A few experienced burnout and ended employment as a result. Reasons for ending employment included the need to recover, regain joy in life, re-discover work satisfaction and take control of the work situation. This decision was sometimes combined with the desire to prioritise family and children.

Making the decision to leave the ICU brought a sense of relief and happiness: ‘*I almost danced out of the ICU (…), it was just a big relief, I do not need to handle all this any longer, at least for a while…*’ (N9). The decision felt right, and few expressed any regrets. However, despite these positive feelings, some referred to the loss of their anticipated professional future. The identity and pride of being an ICU nurse were deeply ingrained. Many had planned a career in intensive care before the pandemic, and there was a sense of grief for the loss of both their professional identity and their expected professional future when transitioning to a different career path:

A future I was prepared for and wanted to have, but then suddenly I didn’t want any more. So, it was like a future that disappeared. A reorientation (N1).

**Fig. 1:**
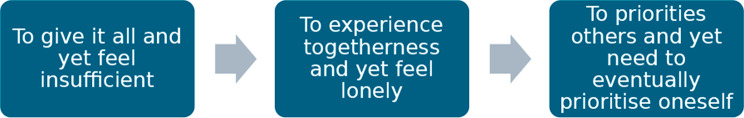
The structural analysis themes: the process to resign

When asked about returning to the ICU in the event of a new pandemic, most expressed a willingness, while a few expressed more ambivalence. Although, many expressed the hope that they would never again have to experience such circumstances, they acknowledged that they would likely return if the situation were to reoccur. Motivators included the need for their unique competence, the opportunity to bring to a similar situation the experience gained from this pandemic and the hope of employing better strategies to handle the situation better next time, ideally based on their own preferences (Fig. 1).

### Comprehensive understanding

Caring for patients and their families during the pandemic was challenging, with sovereign life expressions and ethical demands colliding with the changing cultural norms caused by the circumstances. The nurses were tangled in contradictions and ethical paradoxes where multiple perspectives were experienced simultaneously. Additionally, they were trapped in patients’ stories of suffering, losing their direction and energy when the existential, moral and physical challenges became too great. The initial spontaneous approach to adjust to the situation slowly became more difficult to handle. Although the participants experienced a sense of meaningfulness when caring in a pandemic context, experiences of their own clinical insufficiency collided with spontaneous experiences of mercy and the wish for benevolence. The nurses fought to meet the ethical demands of patients, their families and colleagues, but also of their own families. When practical medical tasks became the prioritised cultural norm, little room was left for compassionate caring. The spontaneous life expressions that were awakened, such as mercy and compassion, were interpreted as being hindered when the relative culture-bearing norms changed and no longer supported the nurses’ experiences and intentions. Those experiences had consequences for both their physical and psychological well-being. Despite their spontaneous willingness to take responsibility for their fellow human beings, the nurses became overwhelmed by the situation, reaching a point where they had to prioritise themselves. This was experienced as a growing need to flee the situation to save oneself. The decision to leave employment in the ICU became vital. The contradictions inherent in the circumstances left the nurses feeling ambivalent, although they ultimately decided—with relief and no regrets, but with sorrow—to give up the fight and flee. At any rate, the decision in its relational and contradictory form was a process. These paradoxes indicate that windows of opportunity may have existed during which these nurses may have been diverted from their course of making the decision to resign. By promoting a supportive culture, understanding the sovereign life expressions and the ethical demands involved in encounters and interactions between nurses and patients, their families, colleagues and their own families, the outcome of the process towards the decision to resign may have been challenged. Further, supporting nurses’ willingness to care and take responsibility for their fellow human beings in crises, confirming nurses’ suffering and their efforts, and considering their individual needs may have reversed their decision to quit.

## Discussion

This study explored ICU nurses’ course towards making the decision to resign from work following the COVID-19 pandemic. The findings described a process in which the nurses were tangled in paradoxes and in which multiple perspectives were experienced simultaneously. The nurses showed a great willingness to work hard and care for patients and their families. At the same time, although they assented to their sovereign life expressions of compassion, trust and mercy when fighting together to fulfil the ethical demands [21, 22] arising from the situation, they felt insufficient and lonely.

The nurses’ spontaneous self-evident willingness to care for patients during the pandemic was initiated by sovereign life expressions such as compassion and mercy for their fellow human beings, which prompted the nurses to work hard despite risks to their health and lives, as shown in a previous study [2]. In line with these findings, a systematic review [24] described nurses’ moral character during the pandemic as being one of professional commitment motivated by an altruistic and professional responsibility. They then experienced ambivalent emotions when confronted with both the patients’ suffering as well as their own vulnerability [24]. Our study shows how nurses’ spontaneous actions were hindered by the demands of the pandemic situation and the conditions for caring when patient survival and medical tasks were prioritised in a stressful environment. During the pandemic, nurses needed to handle a significant number of ethical challenges; they faced threats to the patient and their families dignity, priorities, and in some cases experienced uncertainty or lack of awareness about ethical problems, all while feeling uncomfortable in the situation [24].

The nurses were forced to act in ways contrary to their moral compass, and this left them unable to follow their desired spontaneous, altruistic expressions and actions for their fellow human beings. When the nurses became too exhausted, their sovereign life expressions and willingness to do good for others could no longer carry them, and the nurses became aware of their own circumstances. The nurses’ narratives described experiences of distancing themselves to handle the stressful and ethically challenging situation. According to Martinsen [22], this distancing results in a subsequent ethical dilemma between the moral willingness to do good and the demanded need to act expeditiously. This may also lead to feelings of shame for not being able to live up to one’s morals, and guilt over not being ‘good enough’ when unable to provide the needed care and support for one’s fellow humans, and this may have contributed to their eventual desolation. Such expressions are in line with Martinsen’s [22] thoughts about losing direction and joy when sovereign life expressions are hindered.

Ethics grow out of the spontaneous benevolence that life offers through sovereign spontaneous life expressions, which causes us to forget about ourselves when we engage with our fellow human beings. Martinsen states [22] that fantasy is an important phenomenon in understanding our responsibility towards others. Following routines and regulations due to the crisis, the nurses did what they were obliged to do but were unable to give the desired care. Sovereign life expressions such as mercy and trust or response to ethical demands cannot be followed through routines and regulations. However, with the support of insight, fantasy and understanding it becomes achievable [22]. One negotiable factor is that the culture and cultural-bearing norms that failed to fully support these nurses in their workplace could be of great importance, giving nurses opportunities and a foundation from which to act.

Tangled in paradoxes while showing great willingness to work hard, the nurses felt insufficient and lonely despite experiencing great togetherness; even if they fought hard for their fellow human beings, in the end they felt the need to prioritise themselves and flee. They share with nurses in other studies [24, 25] the effects on the physical, psychological and personal aspects of their lives, their lack of recovery, their exclusion from participating in their work situation and being unable to perform compassionate care, or the desired care, along with the sovereign life expressions. Those experiences pushed the nurses forward on the path towards the decision, however ambivalent they felt, that needed to be made: to stay or to flee. Further, when tangled in contradictory and ethical paradoxes with multiple perspectives experienced simultaneously, the lack of a supportive culture contributed to their final decision to resign and leave the ICU.

Important challenges such as poor relationships and communication with managers, a lack of emotional support, the need to handle moral dilemmas, and personal fatigue and stress have been reported previously to affect the wellbeing of nurses in crisis situations [26]. Intentions to resign from work in relation to a pandemic can be influenced by personal and organisational factors [27]. The question of why nurses resign from ICUs was raised prior to the pandemic [14]. The study showed that both professional reasons, such as workload and rhythm of work, and personal reasons, such as a willingness for change or personal motivation, are the factors most frequently reported by those deciding to leave the ICU. Surprisingly, the COVID-19 pandemic was shown to be associated with nurses remaining in ICU in some clinics, and with resigning in others [14]. A deeper understanding of these differences would be an interesting and important topic for further exploration, including in relation to previous studies where nurses continued to work in the ICU despite experiencing the same challenges [2, 5]. To support nurses’ willingness to work in a crisis, it is important to establish good communication with managers, enhance the sense of responsibility and confidence among health care workers, support policies that address nurses’ emotional needs, and provide facilities for health care workers and their families [26]. Additionally, support from managers and colleagues [28] and support for ethical decision-making [8] has been shown to be important. These factors were also seen in this study.

### Limitations

To enhance the trustworthiness of this study, we relied on information power [29] and took the aim of the study, its theoretical perspectives, the quality of the data and the qualities of the research team into consideration. The recruitment of study participants was based on snowballing and advertising over a period of eight months. Since no official register of nurses who withdrew from ICU existed, and because regulations in Sweden (General Data Protection Regulation) do not allow managers to share information about nurses who resigned during the pandemic, these were found to be the best possible recruitment methods. This strategy though limited the number of potential participants reached by the information about the study. Recruitment challenges may have prolonged the inclusion period. As the first eight interviews gave rich data and the last three confirmed experiences from the course to make the decision to resign, information power was considered fulfilled [29]. Conducting in-depth interviews allowed access to the nurses’ lived experience narratives, while the nurses found relief in reflecting on their experiences, resulting in rich, high-quality interviews. Most interviews (n = 5) were conducted by telephone, with no visual contact between the interviewer and the participants. The participants were able to choose how the interviews were conducted, indicating that they felt comfortable with the option to conduct the meetings via telephone or Zoom. However, there is the possibility that this technology negatively influenced the quality of the interviews by limiting the interaction between the interviewer and participant. The analysis was conducted by all four authors, first individually and thereafter together. All authors have clinical insight into the phenomena studied and are experienced in conducting qualitative analyses. However, as in all qualitative studies, other possible interpretations of the data cannot be excluded. Further, the transferability of the results to other health care contexts, health crises and countries could have been discussed. However, although the participants came from a broad range of backgrounds and from different hospitals, the challenges they encountered and the paradoxes they described during the process to resign seem to describe phenomena that may occur in, or apply to, other health crises.

## Conclusion

The findings of this study suggest that the ICU nurses’ decision to resign was influenced by a tangle of challenging paradoxes that entailed ambivalence. The course to the decision to resign was an ambivalent and contradictory process, with the final decision to resign from the ICU being made with relief and no regrets, but with sorrow. This process could open up opportunities for nursing managers and the health care organisation to change the course of this decision to resign by establishing a supportive culture that encourages nurses to remain on duty in the ICU, by understanding the sovereign life expressions and the ethical demands involved in encounters and interactions between nurses, patients, their families, colleagues and their own families, and by supporting nurses’ willingness to care and take responsibility for their fellow human beings in crises. Further, the system needs to acknowledge the suffering and efforts of nurses and affirm their individual needs. Studies that may be of future interest could further explore the course to nurses’ decision to resign and investigate the extent to which managers were aware of the nurses’ experiences, including how the organisation can prevent nurses from resigning in relation to crises.

## Electronic supplementary material

Below is the link to the electronic supplementary material.

Supplementary Material 1

## Data Availability

The dataset used and/or analysed during the current study are available from the corresponding author on reasonable request.

## References

[1] Fernandez R, Lord H, Halcomb E, Moxham L, Middleton R, Alananzeh I, et al. Implications for COVID-19: a systematic review of nurses’ experiences of working in acute care hospital settings during a respiratory pandemic. Int J Nurs Stud. 2020;111:103637. 32919358 10.1016/j.ijnurstu.2020.103637 PMC7206441

[2] Slettmyr A, Schandl A, Andermo S, Arman M.Spontaneous ethics in nurses’ willingness to work during a pandemic. Nurs Ethics. 2022;29(5):1293–303. 35559725 10.1177/09697330221085768 PMC9111903

[3] Tingsvik C, Bergman L, Falk A-C, Larsson I-M. Long-term impact of COVID-19 on nursing and care delivery: a national survey among anaesthetic and critical care nurses. Aust Crit Care. 2024;37(5):775–782. 10.1016/j.aucc.2024.02.013 38600008

[4] Tong L-K, Zhu M-X, Wang S-C, Cheong P-L, Van I-K. Nurses who are more willing to participate in the fight against COVID-19: evidence from China. Int J Environ Res Public Health. 2021;18(14):7357. 10.3390/ijerph18147357 PMC8305985 34299810

[5] Slettmyr A, Arman M, Andermo S, Malmberg C, Hällström Å, Hugelius K, et al. Intensive care nurses’ lived experience of altruism and sacrifices during the Covid-19 pandemic: a phenomenological study. J Adv Nurs 2023;79:244–53. 36253939 10.1111/jan.15467 PMC9874568

[6] da Silva FCT, Barbosa CP. The impact of the COVID-19 pandemic in an intensive care unit (ICU): psychiatric symptoms in healthcare professionals. Prog Neuropsychopharmacol Biol Psychiatry. 2021;110:110299. 33716042 10.1016/j.pnpbp.2021.110299 PMC7948677

[7] Kissel KA, Filipek C, Jenkins J. Impact of the COVID-19 pandemic on nurses working in intensive care units: a scoping review. Crit Care Nurse. 2023;43(2):55–63. 36804825 10.4037/ccn2023196

[8] Gurdap Z, Cengiz Z. Compassion fatigue and ethical attitudes in nursing care in intensive care nurses during the COVID-19 pandemic: a cross-sectional study. J Nurs Care Qual. 2023;38(4):312–18. 36917830 10.1097/NCQ.0000000000000702

[9] Toscano F, Tommasi F, Giusino D. Burnout in intensive care nurses during the COVID-19 pandemic: a scoping review on its prevalence and risk and protective factors. Int J Environ Res Public Health. 2022;19(12914):12914. 36232211 10.3390/ijerph191912914 PMC9564773

[10] Chen YC, Wu HC, Kuo FT, Koh D, Guo YLL, Shiao JSC. Hospital factors that predict intention of health care workers to leave their job during the COVID-19 pandemic. J Nurs Scholarsh. 2022;54(5):607–12. 35187777 10.1111/jnu.12771 PMC9115187

[11] Chandy P, Kanthi E, Pradeep P, Sathianathan P, Jebakamal S, Narchaithi M, et al. Lived experience of health-care providers during COVID-19: a meta-synthesis. Indian J Psychiatry 2022;64:120. 35494325 10.4103/indianjpsychiatry.indianjpsychiatry_1403_20 PMC9045354

[12] Ebrahimi Rigi Z, Mangolian Shahrbabaki P, Ahmadi F, Ravari A. Self-sacrifice in a distressful and threatening environment: the consequences of the COVID-19 crisis in intensifying workplace violence. Front Psychiatry. 2022;13:848059. 35664478 10.3389/fpsyt.2022.848059 PMC9157344

[13] Xu G, Zeng X, Wu X. Global prevalence of turnover intention among intensive care nurses: a meta-analysis. Nurs Crit Care. 2023;28(2):159–66. 34261191 10.1111/nicc.12679

[14] Vacheron C-H, Bras M, Friggeri A, Manzon C, Vivier E, Caillet A, et al. Factors influencing the turnover of nurses in French intensive care unit-A multicenter interview survey. Anaesthesia Crit Care Pain Med 2025;44:101460. 10.1016/j.accpm.2024.101460 39710228

[15] Lindseth A, Norberg A. A phenomenological hermeneutical method for researching lived experience. Scand J Caring Sci. 2004;18:145–53. 15147477 10.1111/j.1471-6712.2004.00258.x

[16] Lindseth A, Norberg A. Elucidating the meaning of life world phenomena. A phenomenological hermeneutical method for researching lived experience. Scand J Caring Sci. 2022;36(3):883–90. 34687247 10.1111/scs.13039

[17] Charalambous A, Papadopoulos IR, Beadsmoore A.Ricoeur’s hermeneutic phenomenology: an implication for nursing research. Scand J Caring Sci. 2008;22(4):637–42. 19000085 10.1111/j.1471-6712.2007.00566.x

[18] World Medical Association. WMA declaration of Helsinki—ethical principles for medical research involving human subjects 2013 [updated 9th of July 2018]. Available from: https://www.wma.net/policies-post/wma-declaration-of-helsinki-ethical-principles-for-medical-research-involving-human-subjects/. 10.1001/jama.2013.281053 24141714

[19] Tong A, Sainsbury P, Craig J. Consolidated criteria for reporting qualitative research (COREQ): a 32-item checklist for interviews and focus groups. Int J Qual Health Care. 2007;19(6):349–57. 17872937 10.1093/intqhc/mzm042

[20] Løgstrup KE. The Ethical Demand. Notre Dame: University Notre Dame; 1997.

[21] Martinsen K. Care and Vulnerability. Oslo: Akribe; 2006.

[22] Martinsen K. Løgstrup Og Sykepleien. Oslo: Akribe; 2012.

[23] Martinsen K. Langsomme Pulsslag. Bergen: Fagboksforlaget; 2021.

[24] Oh Y, Gastmans C. Ethical issues experienced by nurses during COVID-19 pandemic: systematic review. Nurs Ethics. 2024;31(4):521–40. 37793022 10.1177/09697330231200564

[25] Raso R, Fitzpatrick JJ, Masick K. Nurses’ intent to leave their position and the profession during the COVID-19 pandemic. J Nurs Adm. 2021;51(10):488–94. 34519700 10.1097/NNA.0000000000001052

[26] Nafar H, Tahmazi Aghdam E, Derakhshani N, Sani’ee N, Sharifian S, Goharinezhad S. A systematic mapping review of factors associated with willingness to work under emergency condition. Hum Resources Health. 2021;19(1):76. 10.1186/s12960-021-00622-y PMC8222953 34167560

[27] Varasteh S, Esmaeili M, Mazaheri M.Factors affecting Iranian nurses’ intention to leave or stay in the profession during the COVID-19 pandemic. Int Nurs Rev. 2022;69(2):139–49. 34561862 10.1111/inr.12718 PMC8653279

[28] Toscano F, Tommasi F, Giusino D. Burnout in intensive care nurses during the COVID-19 pandemic: a scoping review on its prevalence and risk and protective factors. Int J Environ Res Public Health. 2022;19(19):12914. 10.3390/ijerph191912914 PMC9564773 36232211

[29] Malterud K, Siersma VD, Guassora AD. Sample size in qualitative interview studies: guided by information power. Qual Health Res. 2016;26(13):1753–60. 26613970 10.1177/1049732315617444

