# Supplementary material for: Fight or flight—intensive care nurses’ decisions to resign following the COVID-19 pandemic: a phenomenological hermeneutical study
Source: BMC Nurs. 2025 Apr 1;24:360. doi: 10.1186/s12912-025-02956-7 (PMC11963475; doi:10.1186/s12912-025-02956-7)
Supplement: Supplementary file 1 — Supplementary Material 1 [file 12912_2025_2956_MOESM1_ESM.docx]

**The interview guide**

Could you describe your experiences working during the pandemic and what led to your decision to resign from your position in the intensive care unit?

What factors influenced your working situation during this period?

Can you share your thoughts and feelings related to your decision to resign?

Did you feel any pressure or demands to continue working in intensive care? If so, from whom and in what way?

Was there anything that could have changed the outcome of your decision?

If you could give three pieces of advice to future colleagues facing a pandemic, what would they be?
